# Supplementary material for: Treatment, Persistent Symptoms, and Depression in People Infected with COVID-19 in Bangladesh
Source: Int J Environ Res Public Health. 2021 Feb 5;18(4):1453. doi: 10.3390/ijerph18041453 (PMC7914967; doi:10.3390/ijerph18041453)
Supplement: Supplementary file 1 [file ijerph-18-01453-s001.zip › ijerph-1068083-supplementary.docx]

List of volunteers who contributed to data collection

| Name | Institution | Email address |
| --- | --- | --- |
| Md. Ahsan Habib | Jashore University of Science & Technology | ahsanhabib.phar@gmail.com |
| Jakia Akter | East West University | pharmasmrity@gmail.com |
| Nasrin Bahadur Nupur | Colonel Malek Medical College | nasrinbahadur25@gmail.com |
| Anika Tahsin Barnana | Home Economics College | barnanasb@gmail.com |
| Rupa Akter | Independent University Bangladesh | akterrupa919@gmail.com |
| Md Delwar Hossen | Independent University, Bangladesh | delwarhossen1097@yahoo.com |
| Mustakim Moyeed | Bangladesh University of Engineering and Technology | mustakimjoy003@gmail.com |
| Hridita Pal | Noakhali Science and Technology University | hridi.pal89@gmail.com |
| Tanzin Nahar | Cumilla Medical College | dr.tanzinnahar@gmail.com |
| Marufa Akter Mim | University of Rajshahi | marufameem94@gmail.com |
| Shanaz Akther | Noakhali Science and Technology University | Shahnazkona99@gmail.com |
| Asif Haque | Noakhali Science and Technology University | haquea432@gmail.com |
| Tasmim Hoq | Independent University Bangladesh | tasmimhoque789@gmail.com |
| Md. Mosaraf Hossain | Jahangirnagar University | hmosarof33@gmail.com |
| Sumona Sarker | Varendra University | s.sarker0103@gmail.com |
| Sadman Sarar | Patuakhali Science and Technology University | sadmansarar777@gmail.com |
| Tanjina Akter Shamme | Jagannath University | angelshammi78@gmail.com |
| Mitu Datto | Patuakhali Science and Technology University | dattomitu123@gmail.com |
| Subrina Farin Prottasha | College of Home Economics, Azimpur | prottasha.uiu@gmail.com |
| Sanjida Akter Riju | Independent University of Bangladesh | rijusanji@gmail.com |
| Tarif Ibne Kamal | Patuakhali Science & Technology University | tarifibnekamal.pstu@gmail.com |
| Nusaiba Jahan | Noakhali science and technology university | nusaibajahannstu@gmail.com |
| Tanvir Ahamed | Bangabandhu Sheikh Mujibur Rahman Science & Technology Universit, Gopalgonj | ttanvirahamed416@gmail.com |
| Kazi Rashadul Islam Rony | National University of Bangladesh (NUB) | rashadul87islam@gmail.com |
| Most. Shamima Akter | University of Rajshahi | shamimaaakter47@gmail.com |
| Md. Shawkat Alam Faisal | University of Rajshahi | shawkat786alam@gmail.com |
| Md. Fakhrul Islam Maruf | Jahangirnagar University | maruf151544@gmail.com |
| Md. Mojammal Huq Bhuiyan | Jahangirnagar University | mojammalb1851@gmail.com |
| Md. Jahidul Islam Sakib | University of Dhaka | jahidsakib171198@gmail.com |
| Tanjina Yousuf | Noakhali Science and Technology University | tanjinayousufjemi@gmail.com |
| Pranta Dhar | Islamic University of Bangladesh | shivushantu36@gmail.com |
| Nyeem Ahamed Khan | North South University | nyem22khan@gmail.com |
| Md Sefatul Islam | Khulna University | 192905@ku.ac.bd |
| Rejina Akter | Jahangirnagar University | Rejinadphi@gmail.com |
| Naimur Rahman | Jahangirnagar University | naimur1046@gmail.com |
| Rashenda Aziz | American Red Cross | mohona.aziz@yahoo.com |
| Khandaker Bushra Rahman | Noakhali Science and Technology University | tashmi0101@gmail.com |
| Sheuli Akter | Bangabandhu Sheikh Mujibur Rahman science and technology university | Sheuliaktersornali27430@gmail.com |
| Ridoy Ahamed | Drug International Limited | ridoy.ahamed@hotmail.com |
| Tasfia Bhuiyan | Noakhali Science & Technology University | tasfiabhuiyan16@gmail.com |
| Kashfiea Nur | College of Home Economics, Azimpur | kashfieanur153@gmail.com |
| Muhammad Al Amin Dewan | Jahangirnagar University | alaminbijoy543026@gmail.com |
| Saifur Rahaman | Noakhali Science and Technology University | saifur.pharma@gmail.com |
| Arifa Akter Tangina | Comilla University | arifaakter7a@gmail.com |
| Tanzila Azad Mow | Jahangirnagar University | azadmow03@gmail.com |
| Mahbuba Alam | Bangladesh University | mahbubaalamsmrity@gmail.com |
